# Integrative transcriptomic analysis revealed the roles and prognostic value of ion channels in hypertrophic cardiomyopathy

**DOI:** 10.3389/fphar.2026.1810143

**Published:** 2026-05-04

**Authors:** Shaohua Li, Jingqi Xu, Feng Zhang, Qing Xu, Donghui Lao, Tianyu Zhang, Yanrong Ye, Yuan Tian

**Affiliations:** 1 Department of Pharmacy, Zhongshan Hospital, Fudan University, Shanghai, China; 2 Department of Facial Plastic Reconstruction Surgery, Eye and ENT Hospital of Fudan University, Shanghai, China; 3 ENT Institute, Eye and ENT Hospital of Fudan University, Shanghai, China; 4 Hearing Medicine Key Laboratory, National Health Commission of China, Shanghai, China; 5 Department of Cardiology, Jinshan Hospital, Fudan University, Shanghai, China

**Keywords:** fibrosis, HCM, ion channel, macrophage, single-cell sequencing, spatial transcriptomics

## Abstract

**Background:**

Hypertrophic cardiomyopathy (HCM) is one of the most common genetic cardiovascular conditions, which may be linked to ion channel dysfunction. In decades, Genetic studies in HCM patients have shown that multiple gene mutations in potassium and calcium ion channels, which may contribute to HCM progression. The direct relationship between HCM and ion channels still needs to be clarified.

**Method:**

We compared the transcriptomic differences between patients with HCM and control populations across three different datasets, and further screened through clustering and enrichment analysis, to identify hub differential expressed ion channels (DEICs) in HCM progression. By constructing pathological features of myocardial hypertrophy, fibrosis, and fibroblast activation, we sought to explore the associations between hub DEICs levels and these pathological features. Subsequently, we used single-cell RNA sequencing (scRNAseq) to clarify the expression characteristics of hub DEICs in different cells and spatial transcriptomic analysis to determine the transcriptomic features of the regions. Based on these hub DEICs, we constructed a prognostic model to evaluate its diagnostic value. Furthermore, we conducted immunoinfiltration analysis to explore their relationship with cardiac inflammatory responses. Finally, we verified these four hub genes expression alteration in transverse aortic constriction (TAC) mice.

**Result:**

Based on three transcriptome sequence datasets, we found 17 ion channels have same expression change in at least two datasets. Trough clustering and enrichment analysis, we narrowed down to four hub DEICs in all datasets, KCNC4, KCNN3, ANO1, CACNA2D3. Additionally, we found that the expression levels of these four genes were significantly associated with myocardial hypertrophy, fibrosis, and fibroblast activation. Further, through scRNAseq, we found that these four genes are primarily expressed in cardiomyocytes. Subsequently, using spatial transcriptomic sequencing, we observed that the regions where these genes were detected exhibited distinct fibrotic characteristics. Based on these hub DEICs, we constructed an expression model and found that their expression levels hold significant diagnostic value for HCM. Additionally, through immunoinfiltration analysis, we identified a correlation between the expression levels of hub DEICs and changes in macrophage proportions. We also found that the changes in gene expression observed in the TAC model were consistent with the aforementioned results.

**Conclusion:**

In this study, through integrative transcriptomic sequencing, we identified four ion channels significantly associated with the development of HCM. Furthermore, in our mechanistic exploration, we found that these associations may be linked to core pathological changes such as myocardial hypertrophy, fibrosis and changes in macrophage proportions.

## Introduction

Hypertrophic cardiomyopathy (HCM) is one of the most common genetic cardiovascular conditions, with an estimated prevalence of one in 200–500 adults (0.2%–0.5%) in the general population worldwide (Semsarian et al., 2015). A practical definition is increased left ventricular wall thickness in the absence of abnormal loading conditions (e.g., hypertension or aortic valve stenosis) capable of stimulating that magnitude of hypertrophy (Ommen et al., 2020). Patients with HCM are more prone to arrhythmias, for example, atrial fibrillation occurs in up to 25% of patients with HCM (Ho et al., 2018). This connection may be linked to ion channel dysfunction (Santini et al., 2021).

HCM is inherited as an autosomal dominant trait in most cases, with offspring having a 50% chance of inheriting the same disease-causing genetic variant (Ommen and Semsarian, 2021). One of the most well recognized functional abnormalities in HCM is dynamic left ventricular outflow tract obstruction (Maron et al., 2006). Typically, patients carry a pathologic DNA variant in genes encoding sarcomere proteins. β-myosin heavy chain (MYH7) and myosin binding protein C3 (MYBPC3) are the genes most commonly involved; however, the causal genes in approximately 40% of patients with HCM remain to be identified (Marian, 2010). Ion channels are closely associated with the occurrence and progression of HCM. *In vitro* experiments have revealed that abnormal expression or enhanced function of calcium channels in cardiomyocytes can activate the calcineurin-NFAT signaling pathway, promoting pathological myocardial hypertrophy (Gao et al., 2012; Coppini et al., 2013). In mouse pressure overload models, the Piezo1 channel perceives mechanical stress and activates hypertrophy-related factors (such as calcineurin) through Ca^2+^ influx, thereby inducing myocardial hypertrophy (Yu et al., 2022). Genetic studies in HCM patients have shown that sequencing has identified multiple gene mutations in potassium and calcium ion channels, which may contribute to disease pathogenesis (Lopes et al., 2013).

By comparing the transcriptomic differences between patients with HCM and normal populations across different datasets, and further screening through clustering and enrichment analysis, we found that four hub DEICs showed significant changes in HCM patients compared to the control group. Subsequently, we used scRNAseq to clarify the expression characteristics of ion channels in different cells and spatial transcriptomic analysis to determine the transcriptomic features of the regions where these hub DEICs were significantly expressed, thereby clarifying their role in the pathological changes of HCM. Furthermore, we conducted immunoinfiltration analysis to explore their relationship with cardiac inflammatory responses. We illustrated that the hub DEICs involved in myocardial hypertrophy, fibrosis and changes in macrophage proportions.

## Methods

### Data acquisition

Three RNA-seq datasets GSE89714 (ref Set1), GSE133054 (ref Set2) (Ren et al., 2020), GSE160997 (ref Set3) (Maron et al., 2021) were downloaded from the GEO database (https://www.ncbi.nlm.nih.gov/geo/). Set1 dataset contained 5 HCM patients and 4 normal samples. Set2 dataset consisted of 8 normal tissue samples and 8 HCM samples. Set 3, however, contains 5 normal samples and 18 HCM samples. Considering the sequencing platform differences and batch effects across datasets, we conduct separate differential analyses for each dataset and then intersect the results for further analysis. Ion channel geneset was downloaded from the HUGO Gene Nomenclature Committee (HGNC) database (https://www.genenames.org/data/genegroup/#!/group/177), and total 330 genes was included in this study.

### Processing of the datasets and screening of DEGs and hub DEICs

The three datasets were translated into the gene symbol by the R package “org.Hs.e.g.,.db” and “org.Mm.e.g.,.db”. Then, differential expressed genes (DEGs) were calculated by R package DESeq2 (Love et al., 2014). Next, DEGs in the HCM patients were screened with cut-off criteria of the false-discovery rate (FDR, Benjamini–Hochberg correction method) <0.05 and |log fold change (FC)| > 1.5. Then, significantly DEGs were identified and separated into upregulated and downregulated subsets. By performing intersection operations with ion channel geneset, significantly DEICs genes were identified.

The four hub genes (KCNC4, KCNN3, ANO1, CACNA2D3) were identified based on two integrated screening strategies. First, in combination with gene enrichment analysis, potassium and calcium channels were highlighted as the most critical ion channel families involved in HCM pathophysiology. Second, these genes were selected because they showed significant differential expression across all three independent datasets.

### Functional enrichment analysis

Gene ontology and signaling pathways associated with DEGs have a significant impact on the development of HCM. Gene Ontology (GO) and Kyoto Encyclopedia of Genes and Genomes (KEGG) pathway analysis were performed by the R package “clusterProfiler” (Wu et al., 2021). FDR <0.05 was considered statistically significant.

### Protein-protein interaction (PPI) network

In this study, we utilized the STRING database (Szklarczyk et al., 2023) (https://cn.string-db.org/) to construct a PPI network for 17 genes of DEICs. The database mapped these genes to their corresponding proteins and generated a PPI network based on known interactions from various sources, including experiments, databases, text mining, and co-expression. Further, we performed a k-means clustering analysis. The clustering algorithm grouped the proteins into three distinct clusters based on their centroids.

### Gene expression and phenotype signature

The expression levels of the four hub DEICs (KCNC4, KCNN3, ANO1, CACNA2D3) were collected across different samples. Genesets for the three phenotypes—myocardial hypertrophy, fibrosis, and fibroblast activation—were obtained. These gene sets were derived from literature review and previous studies. The cardiomyocyte hypertrophy gene set may include genes such as NPPA, NPPB and MYH7, which are known to be upregulated in hypertrophic heart tissue (Ritterhoff et al., 2020; Nomura et al., 2018). The fibrosis gene set might consist of genes like COL1A1, COL1A2 and TGFβ1, which are involved in the synthesis and deposition of extracellular matrix (Yao et al., 2020). The fibroblast activation gene set could include genes such as ACTA2, POSTN and TEAD1, which are markers of activated fibroblasts (Zhang et al., 2020). For each of the four genes, calculate the Pearson correlation coefficient between its expression level and the expression levels of all genes in each of the three phenotype - related gene sets. The Pearson correlation coefficient measures the linear correlation between two variables and ranges from - 1 to 1. Perform t-tests to determine the significance of the correlation coefficients.

### Immune infiltration analysis

We considered widely recognized techniques for assessing immune cell infiltration, including XCELL, TIMER, QUANTISEQ, MCPcounter, EPIC, CIBERSORT-ABS, and CIBERSORT (Xu et al., 2021). Then, we selected macrophage and monocyte proportion to prove the changes between HCM and Control, and was shown in heatmaps. The relationship between gene expression and the differences in macrophage and monocyte proportion investigated using Spearman analysis. Only the results with significant differences were shown in this study.

### ScRNA-seq data preprocessing and analysis

Single-cell RNA sequencing (scRNAseq) data of heart tissues from patients with HCM and controls were downloaded from the GEO database. An integrated analysis was performed after merging the datasets GSE161921, GSE174691 and GSE181764 (Codden and Chin, 2022; Larson et al., 2022). Additionally, single-cell sequencing data of the mouse TAC model were obtained from the GEO database (accession number GSE120064) (Ren et al., 2020). The scRNAseq datasets were processed using Seurat V4 (Hao et al., 2021). For the quality control, cells with fewer than 200 genes and cells with percent of mitochondrial genes over 15% were excluded in this study. For each sample, data normalization and standardization were carried out by means of PCA. The harmony method was utilized to correct for batch effects between samples. The UMAP algorithm was then applied to reduce the dimensionality and visualize the single - cell data. Cells were annotated according to the database CellMarker 2.0 (Hu et al., 2023). The expression level of differential genes in different cell types was displayed by scatter plot.

### Spatial transcriptomics data analysis

Spatial transcriptomics (ST) data of HCM patients were downloaded in Figshare (10.6084/m9. figshare.c.5777948. v2) (Liu et al., 2023). The Seurat R package was used for processing and visualizing the ST data. Data standardization was conducted using the SCT method. Integration of the data was achieved by employing functions like PrepSCTIntegration, FindIntegrationAnchors, SelectIntegrationFeatures, and IntegrateData. Similar ST regions were then grouped through an unsupervised clustering approach. In order to assess gene expression levels in the ST data, both a spatial dim plot and a spatial feature plot were generated. Differential expressed genes were calculated by the functions PreSCTFindMarkers and FindMarkers. GO, KEGG and Gene Set Enrichment Analysis (GSEA) analysis were conducted to enrich the specific cluster or group using the clusterProfiler (Wu et al., 2021). The “h.all. v7.5.2. symbols” gene set from the MSigDB database (https://www.gsea-msigdb.org/gsea/msigdb) was obtained for GSVA analysis to assess the enrichment of different pathways between different samples.

### Construction and evaluation of ion channel genes signature for HCM

The ion channel gene expression signature was then developed by this formula: score = sum (gene expression * correspondence coefficient). Subsequently, we determined all patients’ expression scores using the formula above and divided the patients into high- and low-expression groups based on the numerical values corresponding to their significant inflection points. Then we analyze the differential expressed gene between the high expression group and the expression group, and see the characteristics of the high expression group through GO, KEGG and GSVA enrichment analysis.

### The predictive accuracy of the ion channel genes

To assess the predictive accuracy of the ion channel genes in HCM, receiver operating characteristic (ROC) curves were constructed using the “pROC” package (Robin et al., 2011). The area under the curve (AUC) was calculated as a metric of performance. An AUC value exceeding 0.9 indicated that the hub genes demonstrated a strong predictive capability and a good model fit.

### Establishment of transverse aortic constriction (TAC) model

Eight-week-old adult male C57BL/6 mice were randomly divided into the sham group and the TAC group. The TAC model was established as previously described in the literature (Abuduwufuer et al., 2022). Briefly, mice were anesthetized with isoflurane, followed by a median sternotomy to expose the aorta. A 5–0 braided suture was passed around the aorta, and a 27G needle was used as a template to constrict the aorta. Mice in the sham group underwent the same procedure but without aortic constriction. Subsequently, the sternum and skin of the mice were sutured layer by layer, and the animals were placed on a warming blanket until they regained consciousness and autonomous movement. After 4 weeks of chow diet feeding, all mice were subjected to echocardiographic assessment and tissue harvest. All procedures were approved by the Animal Ethics Committee of Zhongshan Hospital, Fudan University.

### Echocardiographic examination

Mice were anesthetized with isoflurane for induction, then fixed on a warming platform with adhesive tape and maintained under anesthesia with 2% isoflurane inhalation. Echocardiographic data were acquired using a small animal ultrasound imaging system (Vevo 2,100, Fujifilm, VisualSonics, Canada). Cardiac function was evaluated from the left ventricular long-axis view, and the successful establishment of the TAC model was verified by the difference in blood flow velocity observed in the aortic arch view. The imaging protocols were consistent with those described in previous studies (Li et al., 2016).

### RNA extraction and real-time quantitative PCR

Total RNA was extracted from mouse heart tissues using TRIzol reagent (Invitrogen, 15596026). According to the manufacturer’s protocol, RNA was reverse-transcribed into cDNA using a cDNA Synthesis Kit (TaKaRa, RR036A). The resulting cDNA was used as the template for real-time quantitative PCR with TB Green Premix (TaKaRa, RR420A) following the manufacturer’s instructions. Glyceraldehyde-3-phosphate dehydrogenase (GAPDH) was used as the internal reference gene, and the 2^−ΔΔCT^ method was employed to analyze the relative changes in gene expression levels. All primers were designed and synthesized by Sangon Biotech (Shanghai) Co., Ltd.

### Statistical analysis

All statistical analyses were performed using R (version 4.1.2) and GraphPad Prism (version 9.0.0). The correlation between gene and phenotype or gene and cell proportion were investigated using Spearman’s correlation analysis. Wilcoxon test and t-test were used to compare the two groups.

## Results

### Identification of differential expressed ion channels (DEICs) in HCM patients

**FIGURE 1:**
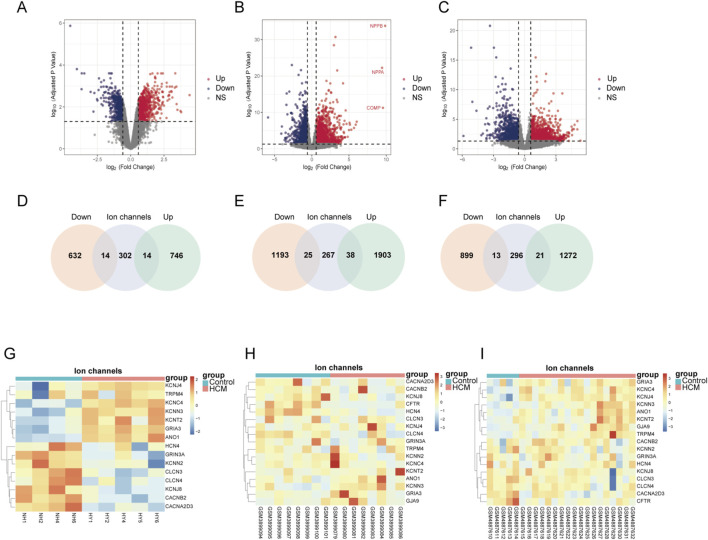
Identification of differential expressed ion channels (DEICs) in HCM patients **(A–C)**, volcano plots of differentially expressed genes across three datasets, where red indicates upregulated genes and blue indicates downregulated genes. **(D–F)**, intersections of upregulated and downregulated genes from the three datasets with anion channel dataset. **(G–I)**, heatmaps showing expression changes of selected ion channels across the three datasets.

To find out significant ion channels in the process of human HCM, we analyzed data obtained from GEO datasets. HCM tissue samples were obtained from patients previously diagnosed with HCM. Control samples were obtained from normal heart donor left ventricles. After normalization, we used DESeq2 to analyse the differential expressed genes in different datasets between HCM patients and healthy people. A total of 760 upregulated DEGs and 646 downregulated DEGs were identified for Set1, with 14 upregulated genes and 14 downregulated genes belonging to ion channels respectively (Figures 1A,D). Similarly, we found 1941 upregulated DEGs and 1,218 downregulated DEGs were identified for Set2, 38 and 25 genes belonging to ion channels respectively (Figures 1B,E), 1,293 upregulated DEGs and 912 downregulated DEGs were identified for Set3, 21 and 13 genes belonging to ion channels respectively (Figures 1C,F). Given the heterogeneity among different datasets, we included ion channels with consistently elevated or reduced expression in at least 2 datasets in our analysis. Ultimately, 17 genes met our criteria and were included. The heatmap revealed that these 17 DEICs exhibited consistent expression patterns across all three datasets (Figures 1G–I), indicating they may play a conserved role in the development of hypertrophic cardiomyopathy.

### DEICs functional analysis and expression differences in HCM

**FIGURE 2:**
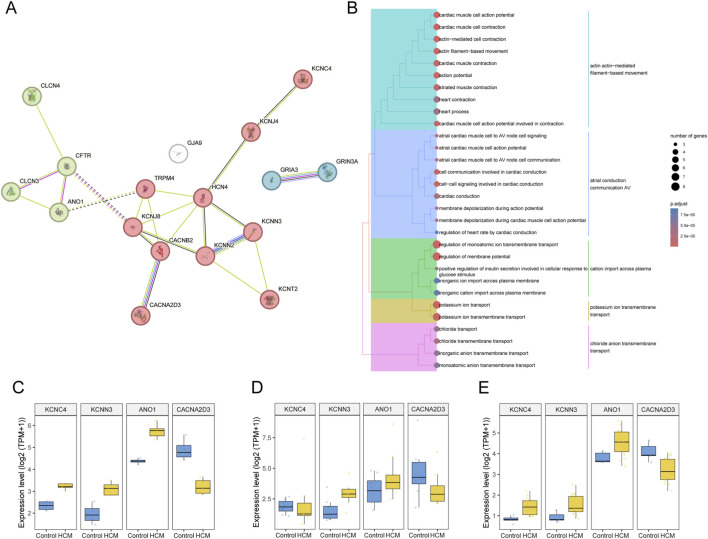
DEICs functional analysis and expression differences in HCM. **(A)** DEICs were used to construct PPI networks, which were subsequently subjected to clustering analysis. **(B)** GO functional enrichment analysis based on DEICs was performed, showcasing the top 30 pathways in the biological process category, with results organized by functional similarity through hierarchical clustering. **(C–E)** expression level variations of filtered hub DEICs across three distinct gene sets are illustrated.

17 DEICs genes were used to construct PPI network, and clustered by k-means algorithm (Figure 2A). We found that these genes were mainly grouped into three clusters. Among them, the most significant were the two clusters dominated by potassium channels (red cluster) and calcium channels (green cluster). Furthermore, we performed GO enrichment analysis on DEICs. GO enrichment also revealed that DEICs in the biological process (BP) were mainly enriched in the potassium and chloride ion transmembrane transport (Figure 2B). Based on these results, we focused on the common potassium channels and calcium channels in these three datasets. We found that 4 genes changed significantly in all of three databases, KCNC4, KCNN3, ANO1, CACNA2D3. The expression of genes KCNC4, KCNN3 and ANO1 significantly increased in HCM group compared to normal group. Conversely, the expression of genes CACNA2D3 significantly decreased (Figures 2C–E).

### Expression heterogeneity of hub DEICs between normal and HCM patients in cardiac cells

**FIGURE 3:**
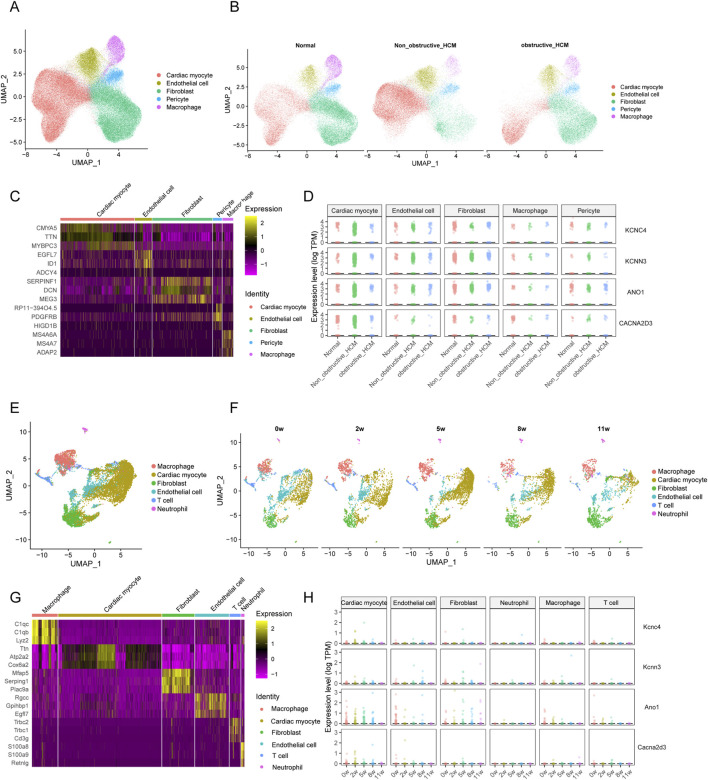
Expression heterogeneity of hub DEICs between normal and HCM patients in cardiac cells. **(A)** Clustering map of dimensionality-reduced and annotated single-cell sequencing data for hypertrophic cardiomyopathy and control populations. **(B)** Distribution of cellular clusters across normal individuals, non-obstructive hypertrophic cardiomyopathy, and obstructive hypertrophic cardiomyopathy. **(C)** Heatmap of expression levels for the top 3 marker genes of each cellular cluster. **(D)** Expression levels of hub DEICs and their changes across different cell types and groups. **(E)** Clustering map of dimensionality-reduced and annotated data from a mouse TAC model at different time points. **(F)** Distribution differences of cellular clusters across modeling time points, grouped by temporal stages. **(G)** Top 3 marker genes for each cellular cluster. **(H)** Differential expression of hub DEICs across cellular clusters and experimental groups.

Owing to the limitation of bulk RNA sequencing in distinguishing gene expression levels among different cell types, we further analyzed scRNAseq data of HCM to clarify the expression differences of these four hub DEICs among various cell types. We integrated three scRNAseq datasets of HCM patients and identified five major cell types based on gene expression differences, including cardiomyocytes, endothelial cells, fibroblasts, pericytes, and macrophages (Figure 3A). Further stratifying the samples based on patient information into normal controls, non-obstructive HCM, and obstructive HCM, we found that all groups contained cells from each category, indicating good control of batch effects among the datasets (Figure 3B). We also plotted marker genes for different cell types, which showed clear distinctions among the cell populations (Figure 3C). Subsequently, we compared the expression levels of these four hub DEICs in different cell types and their trends across the groups, finding that these hub DEICs were predominantly expressed in cardiomyocytes and had higher detection rates in non-obstructive HCM (Figure 3D).

To validate the conclusions regarding differential expression among cell types derived from the single-cell sequencing data, we utilized scRNAseq data from a mouse transverse aortic constriction (TAC) model. The TAC model is a widely accepted model for inducing myocardial hypertrophy in mice and is highly reproducible (Nagalingam et al., 2022). Based on differential gene expression, we classified the cells into macrophages, cardiomyocytes, fibroblasts, endothelial cells, T cells, and neutrophils (Figure 3E). Given the different time points of model induction, we divided the data into five groups representing 0, 2, 5, 8 and 11 weeks post-induction, with all cell types present in each group (Figure 3F). The heatmap of marker genes also demonstrated good distinction in our cell type annotations (Figure 3G). We then compared the expression of these four genes across different cell types and found that they were most highly detected in cardiomyocytes, with the highest detection level for ANO1 (Figure 3H). These results indicate that these four ion channels differentially expressed genes are primarily expressed in cardiomyocytes.

### Relationship between hub DEICs expression and HCM phenotype signature

**FIGURE 4:**
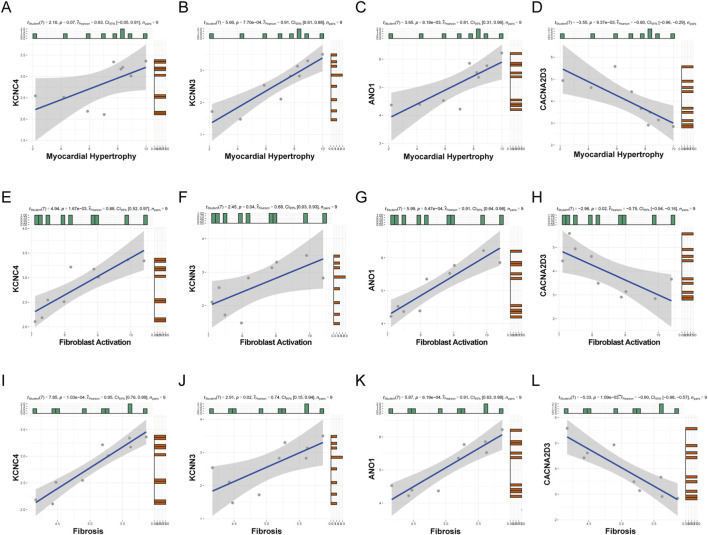
Relationship between hub DEICs expression and HCM phenotype signature. **(A–D)** In Dataset 1, the correlation between hub DEIC expression levels and genes associated with myocardial hypertrophy is illustrated. **(E–H)** In Dataset 1, the relationship between hub DEIC expression levels and genes linked to fibroblast activation is demonstrated. **(I–L)** In Dataset 1, the association between hub DEIC expression levels and genes related to fibrosis is examined.

HCM is a kind of myocardial disease characterized by hypertrophy of the heart, irregular thickening of the heart wall and narrowing of the heart cavity. The pathogenesis of HCM is complicated, which involves the disorder of myocardial fiber arrangement and abnormal morphology (Lillo et al., 2023). Thus, we try to find out the relationship between ion channels and pathological changes. To explore the relationship between these four hub DEICs and the development of HCM, we constructed three pathological phenotypic features of HCM, including myocardial hypertrophy, fibroblast activation, and fibrosis level, and analyzed the correlation between the expression levels of these four hub DEICs and these pathological phenotypes. In Set 1, we found that the expression levels of KCNC4, KCNN3, and ANO1 were significantly positively correlated with myocardial hypertrophy (Figures 4A–C), fibroblast activation (Figures 4E–G), and fibrosis level (Figures 4I–K), with correlation coefficients all above 0.6. In contrast, the expression level of CACNA2D3 was significantly negatively correlated with these three pathological phenotypes (Figures 4D,H,L), with correlation coefficients below −0.75, indicating a strong association. Furthermore, we validated these findings in Set 2 and Set 3 and observed similar trends in most correlations (Supplementary Figures S1A–F). Based on these results, we propose that these four genes are likely involved in the pathophysiological processes of myocardial hypertrophy, fibroblast activation, and fibrosis changes in HCM.

### Spatial distribution characteristics and transcriptomic pattern of DEICs in HCM

**FIGURE 5:**
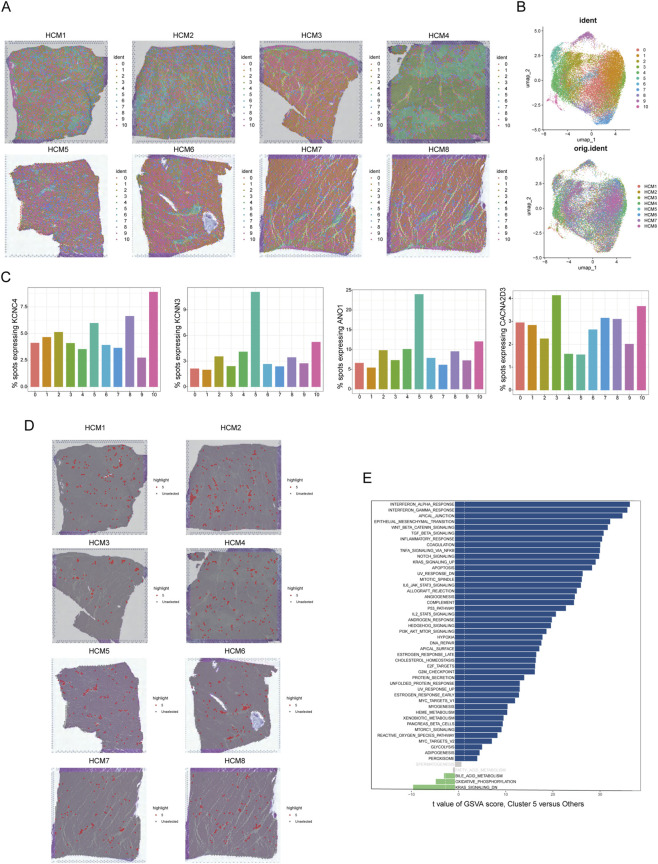
Spatial distribution characteristics of DEICs in HCM. **(A)** Spatial transcriptomic sequencing of cardiac tissues from eight HCM patients was performed. After initial dimensionality reduction and clustering into 11 spot types, their distribution within an H&E-stained image is shown. **(B)**, The distribution of the 11 spot clusters in the dimensionality-reduced space is presented, along with the distribution of each spot type across different samples. **(C)** Changes in the detection rates of hub DEICs for each spot type are illustrated. **(D)** The spatial distribution of cluster 5 is highlighted within the H&E-stained image. **(E)** Significant differences in GSVA pathway enrichment between cluster 5 and other spot clusters are compared.

Given that scRNAseq fails to retain spatial information of gene expression, we further employed spatial transcriptomics (ST) to explore the spatial distribution patterns of hub DEICs. We processed and analyzed the ST data from eight patients with HCM. Through unsupervised clustering, we divided these data into 11 clusters based on the similarity of transcriptional profiles (Figure 5A). Dimensionality reduction revealed distinct spatial locations for these clusters (Figure 5B upper), and the presence of all clusters across different patient samples indicated successful removal of batch effects (Figure 5B lower; Supplementary Figure S2B). Subsequently, we compared the expression levels of the four hub DEICs across these clusters and found that cluster 5 exhibited significantly higher expression of KCNC4, KCNN3, and ANO1, while CACNA2D3 expression was relatively low (Figure 5C). This finding is consistent with our previous observations of expression changes for these four genes in HCM patients. Further analysis of the spatial distribution of spots expressing these genes showed a scattered pattern, with the highest detection rate for ANO1 (Supplementary Figure S2A). Therefore, we propose that cluster 5 likely represents a cluster significantly associated with the expression changes of these four hub DEICs in HCM pathological changes. Combining this with hematoxylin and eosin (HE) staining results, we found that cluster 5 was more prevalent in regions with severe fibrosis (Figure 5D). To characterize the transcriptional features of this cluster, we conducted gene set variation analysis (GSVA) to compare cluster 5 with other clusters, revealing significantly higher activity of fibrosis pathways (TGF-β), inflammatory response, and apoptosis in cluster 5 compared to other clusters (Figure 5E). Additionally, gene ontology (GO) enrichment analysis with hierarchical clustering of biological pathways showed that cluster 5 was significantly enriched in pathways related to extracellular matrix, fibroblast proliferation and activation, and programmed cell death (Supplementary Figure S2C).

**FIGURE 6:**
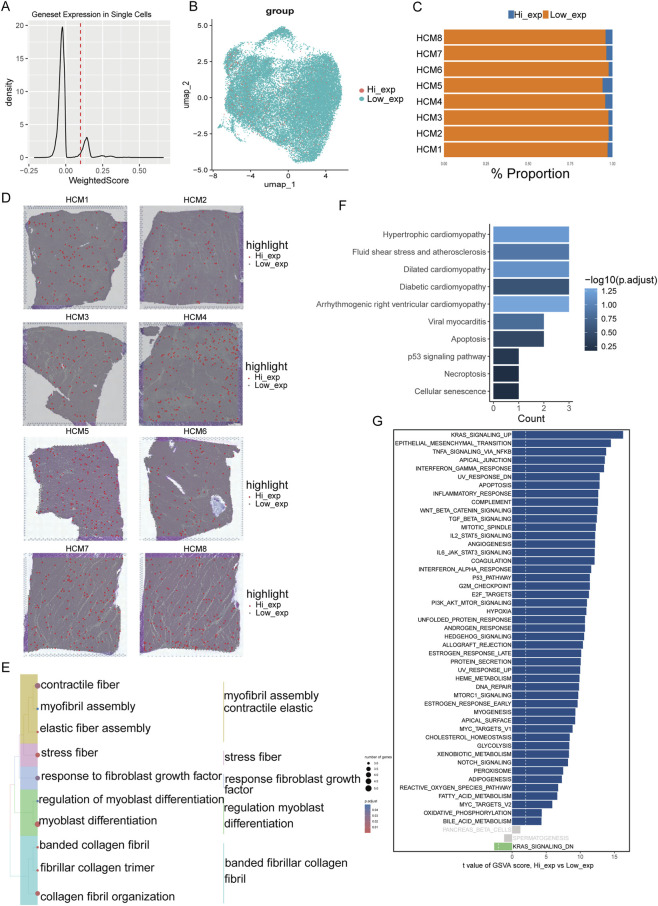
Transcriptomic pattern in high DEICs expression spots in HCM. **(A)** Gene set expression patterns were constructed based on hub DEIC expression levels, with calculations performed to quantify hub DEIC gene set expression in each spot, distinguishing spots with high versus low expression. **(B)** t-SNE maps showing the distribution of spots with high versus low hub DEIC expression after dimensionality reduction and clustering. **(C)** Variations in the proportion of spots with high and low hub DEIC expression across different samples. **(D)** Spatial distribution characteristics of spots with high hub DEIC expression highlighted within H&E-stained images. **(E)** Selected pathways from GO enrichment analysis in the biological process category for spots with high hub DEIC expression. **(F)** KEGG pathway enrichment results for spots exhibiting high hub DEIC expression. **(G)** GSVA pathway differences associated with spots showing high hub DEIC expression.

Next, we performed supervised classification on the ST data. We constructed a geneset based on the expression levels of these four genes, assigning positive weights to the expression levels of KCNC4, KCNN3, and ANO1, which are positively correlated, and negative weights to the expression level of CACNA2D3, which is negatively correlated. We calculated the overall expression levels of these four genes for each spot and generated an expression level map (Figure 6A). Based on this geneset expression level, the spots were divided into high and low expression groups with 0.1 as the threshold. Dimensionality reduction showed that the high-expression group spots were scattered without significant clustering (Figure 6B). Moreover, high-expression group spots were detected in each sample (Figure 6C). Further analysis of the distribution characteristics and transcriptional enrichment features of the high-expression group revealed that, similar to cluster 5, this group also tended to be located in regions with higher fibrosis levels (Figure 6D). GO enrichment analysis results also indicated significant enrichment of the high-expression group in pathways related to extracellular matrix formation and fibroblast proliferation and differentiation (Figure 6E). Additionally, KEGG enrichment analysis showed that the high-expression group was significantly enriched in pathways related to cardiomyopathies such as HCM, dilated cardiomyopathy, diabetic cardiomyopathy, and arrhythmogenic right ventricular cardiomyopathy, as well as in pathways related to apoptosis and necrosis (Figure 6F). Similarly, GSVA analysis of the high-expression group revealed enrichment in pathways related to inflammatory response, fibrosis, and apoptosis (Figure 6G). In conclusion, based on ST data, through unsupervised clustering and expression level grouping analyses, we have once again confirmed that these four hub DEICs are closely related to the pathological features of HCM, such as cardiac fibrosis and fibroblast activation.

### DEICs expression and disease prediction risk in HCM

Our preliminary results showed that the expression levels of these four genes were significantly associated with myocardial hypertrophy, fibrosis severity, and fibroblast activation in HCM. Further ST data analysis also confirmed that their expression levels correlated with the pathophysiological changes in HCM. We then explored whether the expression levels of these four genes could serve as risk indicators for the development of HCM.

We grouped the samples based on disease and control populations and constructed regression models using the expression levels of these genes in each sample, followed by plotting receiver operating characteristic (ROC) curves. We found that in Set 1 and Set 2, the four genes were significantly associated with HCM diagnoses (Supplementary Figures S3A,B). In Set 3, the expression levels of these four genes were also clearly linked to HCM diagnoses. Due to the larger sample size in this gene set, the curve was smoother, and the results were more representative (Supplementary Figure S3C).

### The relation between DEICs and macrophage

In the ST data mentioned above, we observed that the clusters significantly associated with the expression levels of these four hub DEICs were enriched for inflammation-related pathways. We further investigated the relationship between the expression levels of these four genes and immune-related changes during the development of HCM.

**FIGURE 7:**
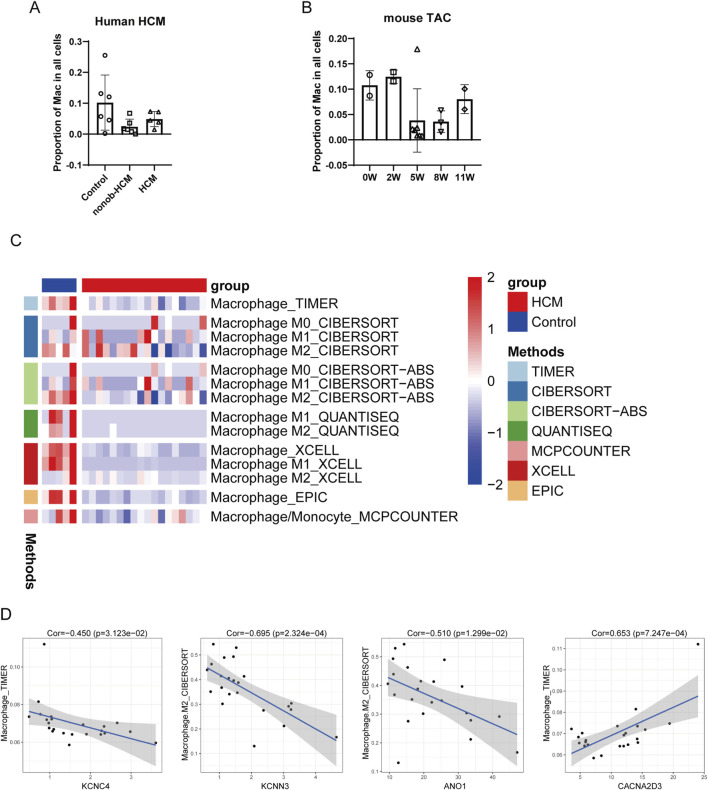
The relation between DEICs and macrophage. **(A)** Changes in the proportion of macrophages in single-cell sequencing of non-obstructive and obstructive HCM patients compared to controls. **(B)** Changes in the proportion of macrophages in cardiac cells of TAC mice with different modeling durations. **(C)** Macrophage proportions in transcriptomic sequencing of HCM patients and controls were calculated using algorithms such as XCELL, TIMER, QUANTISEQ, MCPcounter, EPIC, CIBERSORT-ABS, and CIBERSORT. **(D)** Relationship between hub DEIC expression levels and macrophage proportions.

Based on scRNAseq data, we found that in human HCM datasets, the proportion of macrophages in HCM patients showed a marked decreasing trend compared to the control group (Figure 7A). Similarly, in scRNAseq data from a mouse model of TAC, we observed a reduction in the proportion of macrophages in the hearts of mice 5 weeks after modeling (Figure 7B). To further analyze these changes, we used computational methods, including XCELL, TIMER, QUANTISEQ, MCPcounter, EPIC, CIBERSORT-ABS, and CIBERSORT, to estimate the changes in macrophage proportions between HCM patients and controls in the transcriptomic sequencing data.

We found that in Set 2, the proportion of macrophages in HCM patients was significantly reduced compared to controls across all seven algorithms (Figure 7C). Due to the limited sample size in set 1 and the insufficient number of control subjects in set 3, no statistically significant results were observed. Subsequently, we calculated the correlation between gene expression levels and the cell proportions derived from these algorithms. The results showed that the expression levels of KCNC4, KCNN3, and ANO1 were significantly negatively correlated with macrophage proportions, while the expression level of CACNA2D3 was positively correlated with macrophage proportions (Figure 7D). Based on these findings, we speculate that these genes may also influence the recruitment and infiltration of inflammatory cells in the heart.

### The expression of DEICs in transverse aortic constriction (TAC) model

**FIGURE 8:**
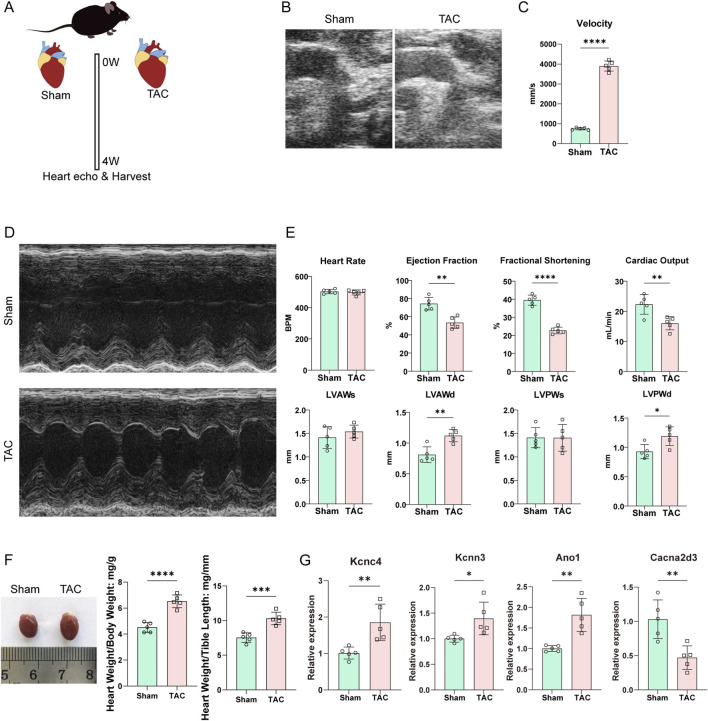
The validation of hub DEICs in HCM. **(A)** The myocardial hypertrophy was induced by transverse aortic constriction (TAC) model for 4 weeks. **(B)** The surgical outcomes of TAC surgery was evaluated through echocardiographic examination. **(C)** The blood flow velocity at the stenotic site in aortic arch of TAC and sham groups. **(D,E)** Effect of TAC surgery on echocardiographic parameters in mice. **(F)** Gross morphological differences in hearts between the two groups are shown: left panel, gross morphology of hearts from the Sham and TAC groups; right panels, ratios of heart weight to body weight and heart weight to tibia length, respectively. ***: p < 0.001. **(G)** The mRNA expression of hub DEICs in TAC and sham groups.

To verify whether the four hub genes we screened were indeed altered during the pathological progression of hypertrophic cardiomyopathy (HCM), we adopted the widely used transverse aortic constriction (TAC) model to induce myocardial hypertrophy. Mice were randomly divided into the sham group and the TAC group, and the animal modeling was conducted for 4 weeks (Figure 8A). Echocardiographic examination of the aortic arch view confirmed that the constriction achieved a satisfactory effect (Figure 8B), with the blood flow velocity at the stenotic site in the TAC group being significantly higher than that in the sham group (Figure 8C). Furthermore, analysis of the left ventricular long-axis view revealed that the cardiac function of mice in the TAC group was significantly impaired compared with the sham group, with decreased ejection fraction and fractional shortening (Figure 8E). Meanwhile, the anterior and posterior wall thicknesses during the diastolic phase in the TAC group were also greater than those in the sham group (Figures 8D,E). At the gross tissue level, a marked cardiac enlargement was clearly observed in the TAC group. Both the heart-to-body weight ratio and the heart-to-tibia length ratio in the TAC group were significantly higher than those in the sham group (Figure 8F). These results indicated that our TAC model successfully induced myocardial hypertrophy.

Based on the established myocardial hypertrophy model, we homogenized the cardiac tissues and extracted total RNA for quantitative PCR analysis. Consistent with our previous findings, the expression of KCNC4, KCNN3 and ANO1 were indeed upregulated in TAC, whereas the expression of CACNA2D3 was downregulated (Figure 8G). Therefore, we concluded that these four genes are core genes involved in the pathological changes of myocardial hypertrophy.

## Discussion

In this study, we focused on the role of ion channels in HCM. By analyzing three transcriptome sequencing datasets, we identified four hub DEICs (KCNC4, KCNN3, ANO1, and CACNA2D3) that may play important roles in HCM. Further validation using scRNAseq data revealed that these ion channels are predominantly expressed in cardiomyocytes. Correlation analyses with three pathological phenotypes—myocardial hypertrophy, fibrosis, and fibroblast activation—showed that KCNC4, KCNN3, and ANO1 were significantly positively correlated with these phenotypes, whereas CACNA2D3 exhibited a negative correlation. Additionally, ST data analysis indicated that the spot clusters with significant changes in gene expression were enriched in fibrosis and inflammation-related pathways. The expression patterns constructed based on these four genes showed a significant increase in fibrosis and inflammation pathways in the high-expression clusters. The expression levels of these four genes also demonstrated high predictive value for HCM. Interestingly, we observed a significant decrease in the proportion of cardiac macrophages during the development of HCM. Using various algorithms to analyze the transcriptome sequencing data of HCM patients, we similarly found a reduction in the proportion of macrophages in HCM patients, and the expression levels of these four genes were significantly associated with the proportion of macrophages. Finally, we verified these four hub genes expression alteration in TAC mice.

Over the past decade, multiple high-throughput sequencing studies have identified changes in ion channel genes in HCM patients, primarily involving potassium and calcium channels (Lopes et al., 2013; Biswas et al., 2019; Krishnaswamy et al., 2024; Hedley et al., 2011; Guo et al., 2017). In rat and mouse models of myocardial hypertrophy, RNA sequencing has also revealed significant alterations in potassium, sodium, and calcium ion channels (Gams et al., 2022; Lu et al., 2018). Common ion channel changes in HCM include alterations in calcium currents and calcium ion concentrations, as well as a decrease in potassium ion currents (Santini et al., 2021). Late depolarization sodium currents can affect the calcium homeostasis of HCM cardiomyocytes via CaMKII, thereby influencing the contraction and relaxation functions of cardiomyocytes (Coppini et al., 2013). Increased expression of polycystic kidney disease-related protein 2-like 1 (PKD2L1) in cardiomyocytes leads to imbalances in intracellular sodium and calcium ions. Elevated intracellular calcium ion concentrations can cause calcium overload in mitochondria, ultimately leading to cardiomyocyte hypertrophy and dysfunction (Lu et al., 2018). In myocardial hypertrophy models, JOSD2 can affect the uptake of calcium ions from the cytoplasm by SERCA2a on the sarcoplasmic reticulum, thereby promoting cardiomyocyte hypertrophy (Han et al., 2023). Additionally, calcium ions can influence pathways such as TNFa/NF-Κb/NLRP3 through calcineurin, regulating inflammation levels in myocardial hypertrophy (Gao et al., 2024). Mechanosensitive cation channel Piezo1, which is involved in mechanotransduction and Ca^2+^ signaling in various cells, plays a crucial role in maintaining intracellular calcium homeostasis. Mice overexpressing Piezo1 show significant increases in cardiac hypertrophy indicators, while Piezo1 knockout significantly inhibits TAC-induced myocardial hypertrophy (Jiang et al., 2021; Zhang et al., 2021). These studies demonstrate that ion channels play important roles in myocardial hypertrophy. Moreover, in HCM patients, a significant correlation has been found between the degree of cardiac fibrosis and cardiac depolarization activity through the combination of electrocardiography and cardiac magnetic resonance imaging, suggesting a potential link between fibrosis and ion channel function in cardiomyocytes (Hurtado-de-Mendoza et al., 2017). Previous studies have also shown that ion channels are associated with fibroblast activation and fibrosis (Abraham et al., 2018). For example, the potassium channel TREK1 activates the JNK pathway in models with increased afterload, contributing to fibroblast activation. Three TRP mechanosensitive ion channels—ATRPC6, TRPM7, and TRPV4—play important roles in fibroblast activation and myocardial fibrosis (Davis et al., 2012; Du et al., 2010; Adapala et al., 2013). Overexpression of Piezo1 in cardiomyocytes leads to significant increases in cardiac fibrosis, while knockout results in a marked reduction in fibrosis levels (Jiang et al., 2021). These findings indicate that ion channels in cardiomyocytes significantly participate in the development of myocardial hypertrophy, fibroblast activation, fibrosis, and inflammation in HCM, providing a theoretical basis for our research results. Based on this, we propose four ion channels that may be associated with the pathogenesis and development of HCM, offering new research directions and ideas for this field.

Ion channels are critically essential for cardiac physiological function. Given that ion channels govern cardiac electrophysiological activities, the dysregulation of ion channel function can trigger abnormal cardiac electrical excitation, thereby resulting in arrhythmias (Crespo-García et al., 2022). Accumulating evidence has demonstrated that multiple ion channels, including PIEZO channels, TRP channels and potassium channels, are closely associated with cardiac fibrosis. Nevertheless, among these investigated channels, only a handful of corresponding agonists and antagonists have been applied in vivo studies, and the research progress of the majority of ion channels relies on gene knockdown or silencing in specific cell types (Wilczak et al., 2025). Pharmacological investigations on ion channels are predominantly dependent on *in vitro* cellular experiments. For instance, 1-EBIO and NS309 are widely utilized to explore the function of small-conductance Ca^2+^-activated K^+^ (SK) channels (Hougaard et al., 2007), and sea anemone toxins serve as essential tools for studying Kv3 potassium channel activity (Yeung et al., 2005). Notably, significant pharmacological discrepancies may emerge in vivo research due to differences in targeted cell populations. Mechanistically, the activation of TRPV1 in cardiomyocytes markedly accelerates cardiomyocyte apoptosis under hypoxic conditions (Wei et al., 2020). By contrast, TRPV1 modulates macrophage polarization in macrophages and further regulates the inflammatory response (Lv et al., 2021). Therefore, achieving precise control of cell selectivity and target specificity remains a major bottleneck that needs to be resolved in cardiac ion channel research.

Regarding the core hub genes screened in the present study, cell-type-specific research has so far only been reported for ANO1. Endothelium-specific knockout of ANO1 significantly reduces blood pressure and ameliorates angiotensin II-induced hypertensive endothelial dysfunction. The underlying mechanism is attributed to the overproduction of reactive oxygen species (ROS), the activation of Nox2-containing NADPH oxidase, and the upregulated protein expression of Nox2 and p22phox (Ma et al., 2017). This mechanistic insight indicates that targeting ANO1 may confer potential therapeutic benefits to patients with hypertrophic cardiomyopathy (HCM). To date, a series of ANO1 modulators, such as T16A inh-A01, niclosamide and CaCCinh-A01, have been identified. However, most of these agents suffer from poor target selectivity and unsatisfactory therapeutic efficacy. Hence, there is an urgent demand for the discovery of novel selective and potent ANO1 modulators for target validation of ANO1 in future translational research (Liu et al., 2021; Genovese et al., 2023).

However, this study also has certain limitations. While the study integrated several transcriptomic data to confirm the role central of ion channels in the progression of HCM, it lacks explicit experimental validation of their associations with multiple pathophysiological processes. Ion channels, as surface proteins on cell membranes, play a critical role in maintaining cellular electrophysiological homeostasis, particularly in cardiomyocytes. However, currently there are no agonists or inhibitors capable of efficiently and selectively targeting these ion channels *in vivo*. The study also lacks corresponding results to validate their specific functions. Future research should focus on screening effective agonists or inhibitors to clarify the specific mechanisms of ion channels in HCM. In the present study, the ROC curve was utilized to preliminarily explore the diagnostic value of these four core genes in patients with HCM. Nevertheless, these findings are merely preliminary bioinformatic results based on the available datasets. Further clinical investigations are still required to validate the clinical diagnostic efficacy of these genes in HCM patients. In addition, experimental verification in this study was only performed using TAC mouse model; subsequent studies should adopt multiple experimental models, including hypertension-induced cardiac hypertrophy, mitral stenosis-induced cardiac hypertrophy, and genetic mutation-induced cardiac hypertrophy, to replicate and validate these conclusions.

## Data Availability

Publicly available datasets were analyzed in this study. This data can be found here: bulk RNA-seq data were obtained from the GEO database (accession numbers: GSE89714, GSE133054, and GSE160997); single-cell transcriptomic sequencing data were downloaded from the GEO database, including GSE161921, GSE174691, GSE181764, and GSE120064; spatial transcriptomic data were retrieved from Figshare (https://doi.org/10.6084/m9.figshare.c.5777948.v2).

## References

[B1] Abraham D. M. Lee T. E. Watson L. J. Mao L. Chandok G. Wang H. G. (2018). The two-pore domain potassium channel TREK-1 mediates cardiac fibrosis and diastolic dysfunction. J. Clin. Invest 128, 4843–4855. 10.1172/JCI95945 30153110 PMC6205385

[B2] Abuduwufuer K. Wang J. J. Li H. Chen C. (2022). A modified technique for transverse aortic constriction in mice. J. Vis. Exp. (186). 10.3791/64386 36063007

[B3] Adapala R. K. Thoppil R. J. Luther D. J. Paruchuri S. Meszaros J. G. Chilian W. M. (2013). TRPV4 channels mediate cardiac fibroblast differentiation by integrating mechanical and soluble signals. J. Mol. Cell Cardiol. 54, 45–52. 10.1016/j.yjmcc.2012.10.016 23142541 PMC3935769

[B4] Biswas A. Raza A. Das S. Kapoor M. Jayarajan R. Verma A. (2019). Loss of function mutation in the P2X7, a ligand-gated ion channel gene associated with hypertrophic cardiomyopathy. Purinergic Signal 15, 205–210. 10.1007/s11302-019-09660-7 31152337 PMC6635509

[B5] Codden C. J. Chin M. T. (2022). Common and distinctive intercellular communication patterns in human obstructive and nonobstructive hypertrophic cardiomyopathy. Int. J. Mol. Sci. 23, 946. 10.3390/ijms23020946 35055131 PMC8780670

[B6] Coppini R. Ferrantini C. Yao L. Fan P. Del Lungo M. Stillitano F. (2013). Late sodium current inhibition reverses electromechanical dysfunction in human hypertrophic cardiomyopathy. Circulation 127, 575–584. 10.1161/CIRCULATIONAHA.112.134932 23271797

[B7] Crespo-García T. Cámara-Checa A. Dago M. Rubio-Alarcón M. Rapún J. Tamargo J. (2022). Regulation of cardiac ion channels by transcription factors: looking for new opportunities of druggable targets for the treatment of arrhythmias. Biochem. Pharmacol. 204, 115206. 10.1016/j.bcp.2022.115206 35963339

[B8] Davis J. Burr A. R. Davis G. F. Birnbaumer L. Molkentin J. D. (2012). A TRPC6-dependent pathway for myofibroblast transdifferentiation and wound healing *in vivo* . Dev. Cell 23, 705–715. 10.1016/j.devcel.2012.08.017 23022034 PMC3505601

[B9] Du J. Xie J. Zhang Z. Tsujikawa H. Fusco D. Silverman D. (2010). TRPM7-mediated Ca2+ signals confer fibrogenesis in human atrial fibrillation. Circ. Res. 106, 992–1003. 10.1161/CIRCRESAHA.109.206771 20075334 PMC2907241

[B10] Gams A. Brennan J. A. Goldrick K. Efimov I. R. (2022). Molecular and functional remodeling of superior and inferior SAN in a rat model of HCM. JACC Clin. Electrophysiol. 8, 1341–1353. 10.1016/j.jacep.2022.08.003 36424000

[B11] Gao H. Wang F. Wang W. Makarewich C. A. Zhang H. Kubo H. (2012). Ca(2+) influx through L-type Ca(2+) channels and transient receptor potential channels activates pathological hypertrophy signaling. J. Mol. Cell Cardiol. 53, 657–667. 10.1016/j.yjmcc.2012.08.005 22921230 PMC3472041

[B12] Gao Y. Li S. Liu X. Si D. Chen W. Yang F. (2024). RyR2 stabilizer attenuates cardiac hypertrophy by downregulating TNF-α/NF-κB/NLRP3 signaling pathway through inhibiting calcineurin. J. Cardiovasc Transl. Res. 17, 481–495. 10.1007/s12265-023-10376-8 38652413

[B13] Genovese M. Buccirossi M. Guidone D. De Cegli R. Sarnataro S. Di Bernardo D. (2023). Analysis of inhibitors of the anoctamin-1 chloride channel (transmembrane member 16A, TMEM16A) reveals indirect mechanisms involving alterations in calcium signalling. Br. J. Pharmacol. 180, 775–785. 10.1111/bph.15995 36444690

[B14] Guo X. Fan C. Tian L. Liu Y. Wang H. Zhao S. (2017). The clinical features, outcomes and genetic characteristics of hypertrophic cardiomyopathy patients with severe right ventricular hypertrophy. PLoS One 12, e0174118. 10.1371/journal.pone.0174118 28323875 PMC5360271

[B15] Han J. Fang Z. Han B. Ye B. Lin W. Jiang Y. (2023). Deubiquitinase JOSD2 improves calcium handling and attenuates cardiac hypertrophy and dysfunction by stabilizing SERCA2a in cardiomyocytes. Nat. Cardiovasc Res. 2, 764–777. 10.1038/s44161-023-00313-y 39195964

[B16] Hao Y. Hao S. Andersen-Nissen E. Mauck W. M. Zheng S. Butler A. (2021). Integrated analysis of multimodal single-cell data. Cell 184, 3573–3587.e29. 10.1016/j.cell.2021.04.048 34062119 PMC8238499

[B17] Hedley P. L. Haundrup O. Andersen P. S. Aidt F. H. Jensen M. Moolman-Smook J. C. (2011). The KCNE genes in hypertrophic cardiomyopathy: a candidate gene study. J. Negat. Results Biomed. 10, 12. 10.1186/1477-5751-10-12 21967835 PMC3204304

[B18] Ho C. Y. Day S. M. Ashley E. A. Michels M. Pereira A. C. Jacoby D. (2018). Genotype and lifetime burden of disease in hypertrophic cardiomyopathy: insights from the sarcomeric human cardiomyopathy registry (SHaRe). Circulation 138, 1387–1398. 10.1161/CIRCULATIONAHA.117.033200 30297972 PMC6170149

[B19] Hougaard C. Eriksen B. L. Jørgensen S. Johansen T. H. Dyhring T. Madsen L. S. (2007). Selective positive modulation of the SK3 and SK2 subtypes of small conductance Ca2+-activated K+ channels. Br. J. Pharmacol. 151, 655–665. 10.1038/sj.bjp.0707281 17486140 PMC2014002

[B20] Hu C. Li T. Xu Y. Zhang X. Li F. Bai J. (2023). CellMarker 2.0: an updated database of manually curated cell markers in human/mouse and web tools based on scRNA-seq data. Nucleic Acids Res. 51, D870–D876. 10.1093/nar/gkac947 36300619 PMC9825416

[B21] Hurtado-De-Mendoza D. Corona-Villalobos C. P. Pozios I. Gonzales J. Soleimanifard Y. Sivalokanathan S. (2017). Diffuse interstitial fibrosis assessed by cardiac magnetic resonance is associated with dispersion of ventricular repolarization in patients with hypertrophic cardiomyopathy. J. Arrhythm. 33, 201–207. 10.1016/j.joa.2016.10.005 28607615 PMC5459419

[B22] Jiang F. Yin K. Wu K. Zhang M. Wang S. Cheng H. (2021). The mechanosensitive Piezo1 channel mediates heart mechano-chemo transduction. Nat. Commun. 12, 869. 10.1038/s41467-021-21178-4 33558521 PMC7870949

[B23] Krishnaswamy S. M. Arunachal G. Singh K. G. Thomson V. S. George P. Rao S. (2024). Investigation of mutation spectrum amongst patients with familial primary cardiomyopathy using targeted NGS in Indian population. J. Appl. Genet. 65, 809–822. 10.1007/s13353-024-00855-2 38551768

[B24] Larson A. Codden C. J. Huggins G. S. Rastegar H. Chen F. Y. Maron B. J. (2022). Altered intercellular communication and extracellular matrix signaling as a potential disease mechanism in human hypertrophic cardiomyopathy. Sci. Rep. 12, 5211. 10.1038/s41598-022-08561-x 35338173 PMC8956620

[B25] Li L. Guo X. Chen Y. Yin H. Li J. Doan J. (2016). Assessment of cardiac morphological and functional changes in mouse model of transverse aortic constriction by echocardiographic imaging. J. Vis. Exp. (112), 54101. 10.3791/54101 27403841 PMC4917259

[B26] Lillo R. Graziani F. Franceschi F. Iannaccone G. Massetti M. Olivotto I. (2023). Inflammation across the spectrum of hypertrophic cardiac phenotypes. Heart Fail Rev. 28, 1065–1075. 10.1007/s10741-023-10307-4 37115472 PMC10403403

[B27] Liu Y. Liu Z. Wang K. (2021). The Ca(2+)-activated chloride channel ANO1/TMEM16A: an emerging therapeutic target for epithelium-originated diseases? Acta Pharm. Sin. B 11, 1412–1433. 10.1016/j.apsb.2020.12.003 34221860 PMC8245819

[B28] Liu X. Yin K. Chen L. Chen W. Li W. Zhang T. (2023). Lineage-specific regulatory changes in hypertrophic cardiomyopathy unraveled by single-nucleus RNA-seq and spatial transcriptomics. Cell Discov. 9, 6. 10.1038/s41421-022-00490-3 36646705 PMC9842679

[B29] Lopes L. R. Zekavati A. Syrris P. Hubank M. Giambartolomei C. Dalageorgou C. (2013). Genetic complexity in hypertrophic cardiomyopathy revealed by high-throughput sequencing. J. Med. Genet. 50, 228–239. 10.1136/jmedgenet-2012-101270 23396983 PMC3607113

[B30] Love M. I. Huber W. Anders S. (2014). Moderated estimation of fold change and dispersion for RNA-seq data with DESeq2. Genome Biol. 15, 550. 10.1186/s13059-014-0550-8 25516281 PMC4302049

[B31] Lu Z. Cui Y. Wei X. Gao P. Zhang H. Wei X. (2018). Deficiency of PKD2L1 (TRPP3) exacerbates pathological cardiac hypertrophy by augmenting NCX1-Mediated mitochondrial calcium overload. Cell Rep. 24, 1639–1652. 10.1016/j.celrep.2018.07.022 30089272

[B32] Lv Z. Xu X. Sun Z. Yang Y. X. Guo H. Li J. (2021). TRPV1 alleviates osteoarthritis by inhibiting M1 macrophage polarization via Ca(2+)/CaMKII/Nrf2 signaling pathway. Cell Death Dis. 12, 504. 10.1038/s41419-021-03792-8 34006826 PMC8131608

[B33] Ma M. M. Gao M. Guo K. M. Wang M. Li X. Y. Zeng X. L. (2017). TMEM16A contributes to endothelial dysfunction by facilitating Nox2 NADPH oxidase-derived reactive oxygen species generation in hypertension. Hypertension 69, 892–901. 10.1161/HYPERTENSIONAHA.116.08874 28320851

[B34] Marian A. J. (2010). Hypertrophic cardiomyopathy: from genetics to treatment. Eur. J. Clin. Invest 40, 360–369. 10.1111/j.1365-2362.2010.02268.x 20503496 PMC2903630

[B35] Maron M. S. Olivotto I. Zenovich A. G. Link M. S. Pandian N. G. Kuvin J. T. (2006). Hypertrophic cardiomyopathy is predominantly a disease of left ventricular outflow tract obstruction. Circulation 114, 2232–2239. 10.1161/CIRCULATIONAHA.106.644682 17088454

[B36] Maron B. A. Wang R. S. Shevtsov S. Drakos S. G. Arons E. Wever-Pinzon O. (2021). Individualized interactomes for network-based precision medicine in hypertrophic cardiomyopathy with implications for other clinical pathophenotypes. Nat. Commun. 12, 873. 10.1038/s41467-021-21146-y 33558530 PMC7870822

[B37] Nagalingam R. S. Chattopadhyaya S. Al-Hattab D. S. Cheung D. Y. C. Schwartz L. Y. Jana S. (2022). Scleraxis and fibrosis in the pressure-overloaded heart. Eur. Heart J. 43, 4739–4750. 10.1093/eurheartj/ehac362 36200607

[B38] Nomura S. Satoh M. Fujita T. Higo T. Sumida T. Ko T. (2018). Cardiomyocyte gene programs encoding morphological and functional signatures in cardiac hypertrophy and failure. Nat. Commun. 9, 4435. 10.1038/s41467-018-06639-7 30375404 PMC6207673

[B39] Ommen S. R. Semsarian C. (2021). Hypertrophic cardiomyopathy: a practical approach to guideline directed management. Lancet 398, 2102–2108. 10.1016/S0140-6736(21)01205-8 34600606

[B40] Ommen S. R. Mital S. Burke M. A. Day S. M. Deswal A. Elliott P. (2020). 2020 AHA/ACC guideline for the diagnosis and treatment of patients with hypertrophic cardiomyopathy: executive summary: a report of the American college of cardiology/american heart association joint committee on clinical practice guidelines. Circulation 142, e533–e557. 10.1161/CIR.0000000000000938 33215938

[B41] Ren Z. Yu P. Li D. Li Z. Liao Y. Wang Y. (2020). Single-cell reconstruction of progression trajectory reveals intervention principles in pathological cardiac hypertrophy. Circulation 141, 1704–1719. 10.1161/CIRCULATIONAHA.119.043053 32098504

[B42] Ritterhoff J. Young S. Villet O. Shao D. Neto F. C. Bettcher L. F. (2020). Metabolic remodeling promotes cardiac hypertrophy by directing glucose to aspartate biosynthesis. Circ. Res. 126, 182–196. 10.1161/CIRCRESAHA.119.315483 31709908 PMC8448129

[B43] Robin X. Turck N. Hainard A. Tiberti N. Lisacek F. Sanchez J. C. (2011). pROC: an open-source package for R and S+ to analyze and compare ROC curves. BMC Bioinforma. 12, 77. 10.1186/1471-2105-12-77 21414208 PMC3068975

[B44] Santini L. Coppini R. Cerbai E. (2021). Ion channel impairment and myofilament Ca(2+) sensitization: two parallel mechanisms underlying arrhythmogenesis in hypertrophic cardiomyopathy. Cells 10, 2789. 10.3390/cells10102789 34685769 PMC8534456

[B45] Semsarian C. Ingles J. Maron M. S. Maron B. J. (2015). New perspectives on the prevalence of hypertrophic cardiomyopathy. J. Am. Coll. Cardiol. 65, 1249–1254. 10.1016/j.jacc.2015.01.019 25814232

[B46] Szklarczyk D. Kirsch R. Koutrouli M. Nastou K. Mehryary F. Hachilif R. (2023). The STRING database in 2023: protein-protein association networks and functional enrichment analyses for any sequenced genome of interest. Nucleic Acids Res. 51, D638–D646. 10.1093/nar/gkac1000 36370105 PMC9825434

[B47] Wei J. Lin J. Zhang J. Tang D. Xiang F. Cui L. (2020). TRPV1 activation mitigates hypoxic injury in mouse cardiomyocytes by inducing autophagy through the AMPK signaling pathway. Am. J. Physiol. Cell Physiol. 318, C1018–c1029. 10.1152/ajpcell.00161.2019 32293932

[B48] Wilczak W. Schleinhege R. Loescher C. M. Schwab A. Pethő Z. (2025). A tough job: ion channels, transporters, and pumps during organ fibrosis. Am. J. Physiol. Cell Physiol. 329, C2091–c2111. 10.1152/ajpcell.00564.2025 41071674

[B49] Wu T. Hu E. Xu S. Chen M. Guo P. Dai Z. (2021). clusterProfiler 4.0: a universal enrichment tool for interpreting omics data. Innov. (Camb) 2, 100141. 10.1016/j.xinn.2021.100141 34557778 PMC8454663

[B50] Xu Q. Xu H. Deng R. Wang Z. Li N. Qi Z. (2021). Multi-omics analysis reveals prognostic value of tumor mutation burden in hepatocellular carcinoma. Cancer Cell Int. 21, 342. 10.1186/s12935-021-02049-w 34217320 PMC8254981

[B51] Yao Y. Hu C. Song Q. Li Y. Da X. Yu Y. (2020). ADAMTS16 activates latent TGF-β, accentuating fibrosis and dysfunction of the pressure-overloaded heart. Cardiovasc Res. 116, 956–969. 10.1093/cvr/cvz187 31297506 PMC7868664

[B52] Yeung S. Y. Thompson D. Wang Z. Fedida D. Robertson B. (2005). Modulation of Kv3 subfamily potassium currents by the sea anemone toxin BDS: significance for CNS and biophysical studies. J. Neurosci. 25, 8735–8745. 10.1523/JNEUROSCI.2119-05.2005 16177043 PMC1314979

[B53] Yu Z. Y. Gong H. Kesteven S. Guo Y. Wu J. Li J. V. (2022). Piezo1 is the cardiac mechanosensor that initiates the cardiomyocyte hypertrophic response to pressure overload in adult mice. Nat. Cardiovasc Res. 1, 577–591. 10.1038/s44161-022-00082-0 39195867 PMC11358016

[B54] Zhang W. Wang L. Lu Z. Wang B. Li Y. Yang J. (2020). Discovery of natural compounds for cardiac fibrosis by a transcriptome-based functional gene module reference approach. J. Nat. Prod. 83, 2923–2930. 10.1021/acs.jnatprod.0c00453 33006888

[B55] Zhang Y. Su S. A. Li W. Ma Y. Shen J. Wang Y. (2021). Piezo1-Mediated mechanotransduction promotes cardiac hypertrophy by impairing calcium homeostasis to activate calpain/calcineurin signaling. Hypertension 78, 647–660. 10.1161/HYPERTENSIONAHA.121.17177 34333987

