# Supplementary material for: Integrative transcriptomic analysis revealed the roles and prognostic value of ion channels in hypertrophic cardiomyopathy
Source: Front Pharmacol. 2026 May 4;17:1810143. doi: 10.3389/fphar.2026.1810143 (PMC13180936; doi:10.3389/fphar.2026.1810143)
Supplement: Supplementary file 1 [file Supplementaryfile1.docx]

**Supplementary Materials**


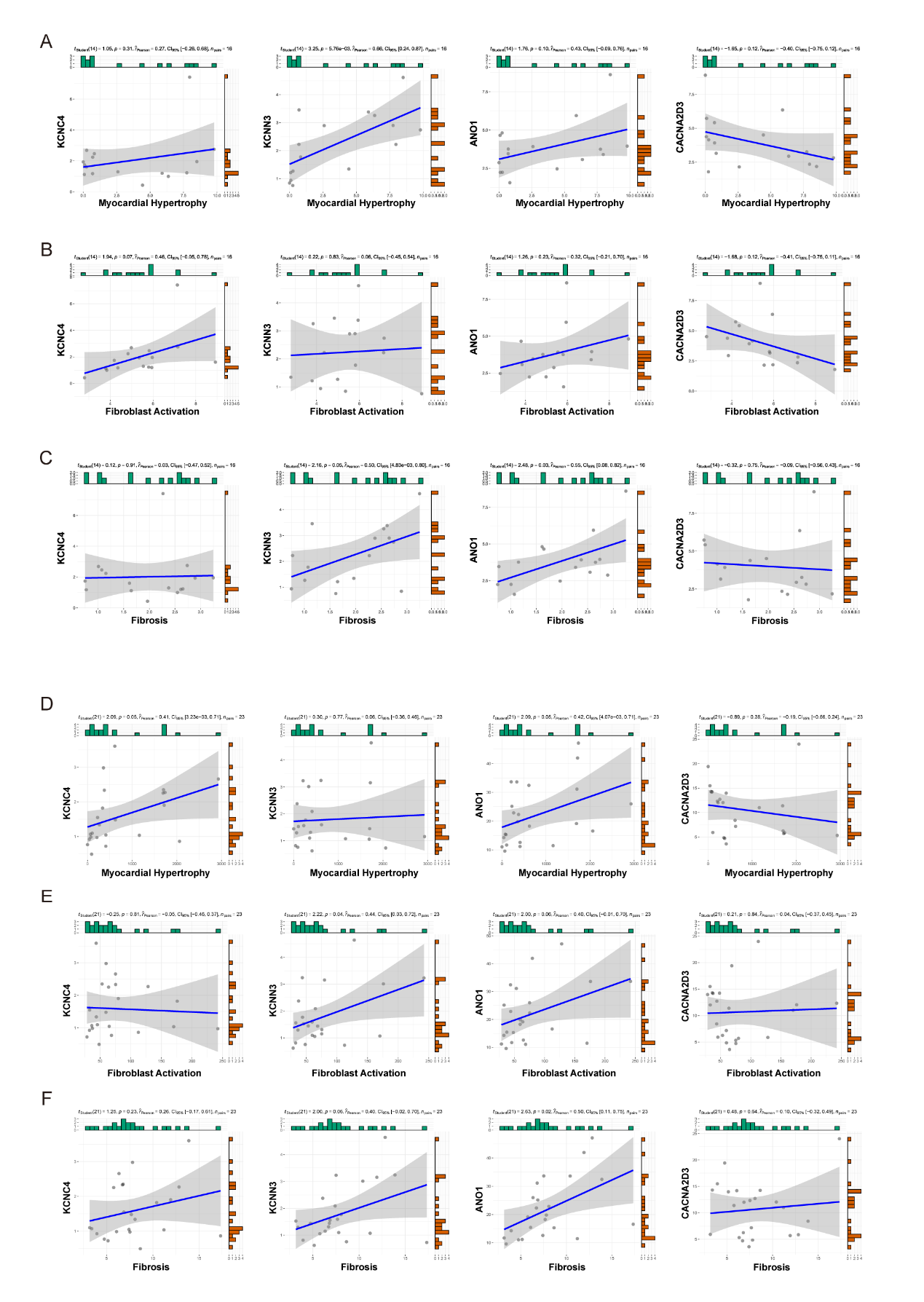


Fig. S1: Relationship between hub DEICs expression and HCM phenotype signature

A-C, In Dataset 2, the correlation between hub DEIC expression levels and the expression levels of genes associated with myocardial hypertrophy, fibroblast activation, and fibrosis is illustrated. D-F, In Dataset 3, the relationship between hub DEIC expression levels and the expression levels of genes linked to myocardial hypertrophy, fibroblast activation, and fibrosis is demonstrated.


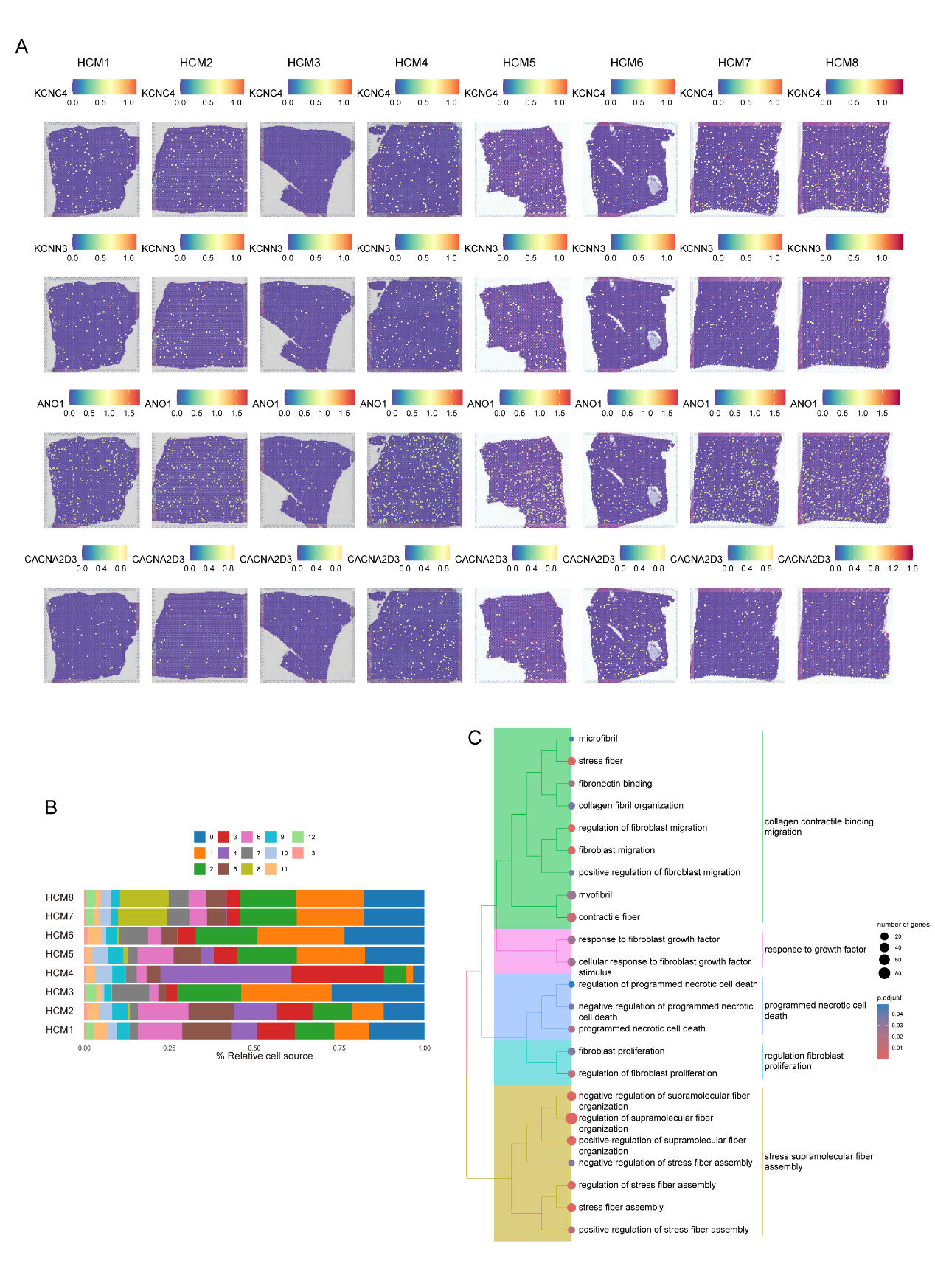


Fig. S2: Basic information of spatial transcriptome of HCM

A, Changes in the expression levels of four hub DEICs across samples are illustrated within H&E-stained images. B, Variations in the proportions of different clusters across samples are presented. C, Key pathways showing significant changes in biological processes from GO enrichment analysis when comparing cluster 5 with other clusters are highlighted. D, The association between hub DEIC expression levels and changes in macrophage proportions is depicted.


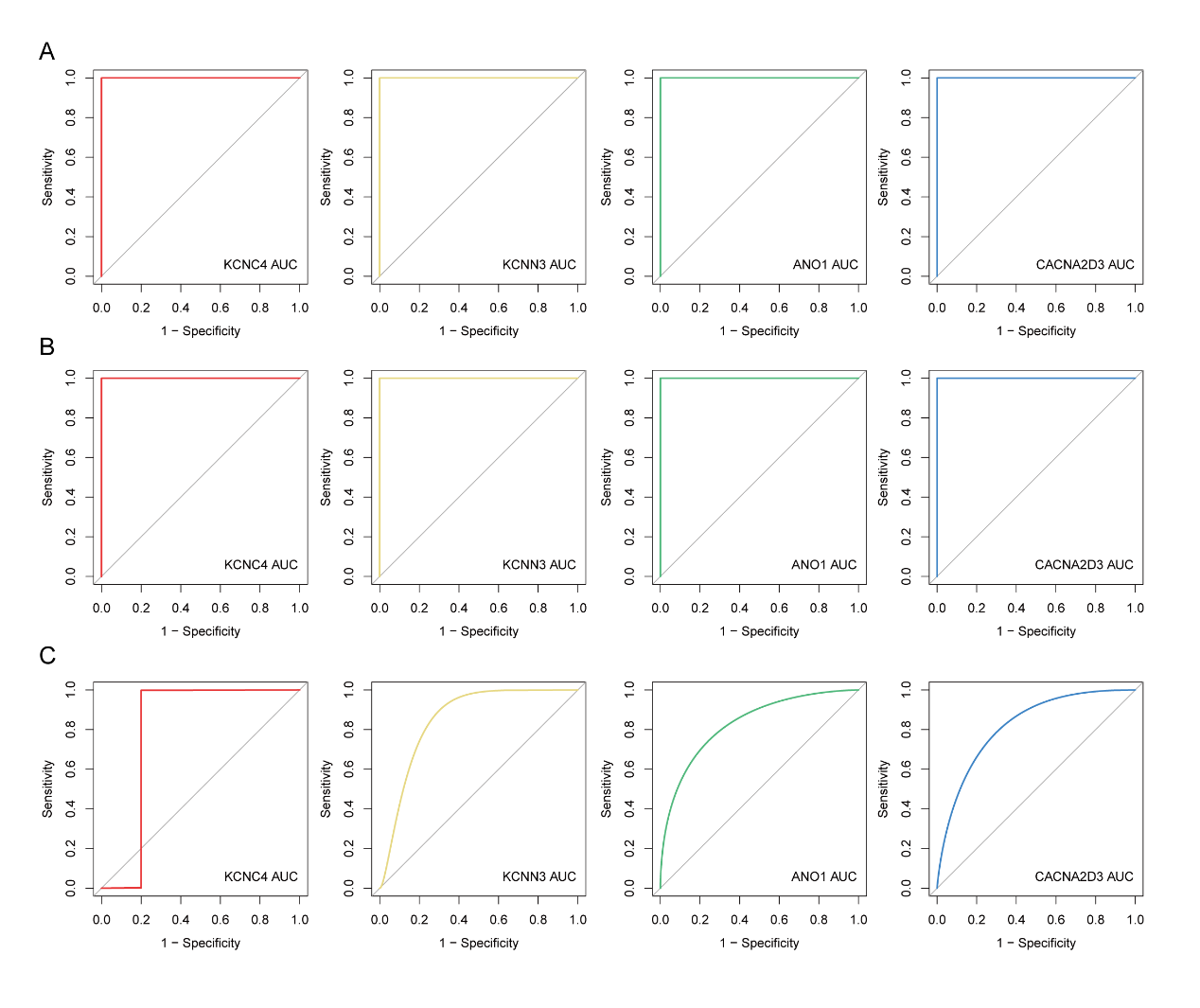


Fig. S3: DEICs Expression and Disease Prediction Risk in HCM

A-C, The diagnostic capability of four hub DEICs for HCM is illustrated via AUC curves in Dataset 1, Dataset 2, and Dataset 3, respectively.
